# 

**Published:** 1900-03

**Authors:** 


### BOOKS RECEIVED.

Report of the Commissioner of Education for the Year 1897-98. Volume 2, containing parts II. and III. 8vo, pp 2640. Washington: Government Printing Office. 1899.

A Practical Treatise on Diseases of the Skin. For the use of Students and Practitioners. By James Nevins Hyde, A. M., M. D., Professor of Dermatology and Venereal Diseases in Rush Medical College, Chicago New (fifth) edition. In one octavo volume of 866 pages, with 111 engravings and 24 full-page plates, eight of which are colored. Cloth, $4.50, net; leather, $5 50, net. Philadelphia: Lea Brothers & Co. 1900.

The American Year-Book of Medicine and Surgery. Edited by George M. Gould, M. D. In two handsome octavo volumes. Profusely illustrated. Cloth, $3.00 per volume, net; half morocco, $3.75 per volume, net. Philadelphia: W. B Saunders, Publisher, 925 Walnut St. 1900.

The Principles of Treatment and Their Application to the Practice of Medicine By J. Mitchell Bruce, M. D., F. R. C. P., Lecturer on the Practice of Medicine in the Charing-Cross Hospital, London ; Examiner in Medicine, Royal College of Physicians, London. Revised to conform with the U. S. Pharmacopeia by E. Q. Thornton, M. D , Jefferson Medical College, Philadelphia. In one octavo volume of 625 pages. Cloth, $3.75. net. Philadelphia : Lea Brothers & Co. 1900.

Transactions of the Mississippi Valley Medical Association. Twenty-fifth Annual Session, held at Chicago, Ill., October 3, 4, 5 and 6, 1899. Volume I. Edited by Henry E. Tuley, M. D., Secretary, Louisville, Ky. 8vo, pp. 474. Louisville, Ky.: The Courier-Journal Print. 1900.

Potts's Nervous and Mental Diseases. A Pocket Text-Book of Nervous and Mental Diseases. By Charles S. Potts, M. D., Instructor in Electro-Therapeutics and Nervous Diseases in the University of Pennsylvania, Philadelphia. In one handsome 12mo volume of 442 pages, with 88 illustrations. Cloth, $1.75 net ; Flexible Red Leather, $2 25, net. Philadelphia and New York : Lea Brothers & Co. February, 1900.

Crockett's Gynecology. A Pocket Text-Book of Diseases of Women. By Montgomery A. Crockett, A. B., M. D., Adjunct Professor of Obstetrics and Clinical Gynecology, Medical Department of the University of Buffalo, N. Y. In one handsome 12mo volume of 368 pages, with 107 illustrations. Cloth, $1.50, net ; Flexible Red Leather, $2.00, net. Philadelphia and New York : Lea Brothers & Co. February, 1900.

Proceedings of the American Medico-Psycological Association at the Fifty-fifth Annual Meeting, held at New York City, May 23, 24, 25 and 26, 1899. Edited by C. B. Burr, M. D., Secretary. Published by the Association. 1900.

